# Value Frameworks: Adaptation of Korean Versions of Value Frameworks for Oncology

**DOI:** 10.3390/ijerph18063139

**Published:** 2021-03-18

**Authors:** Green Bae, SeungJin Bae, Donghwan Lee, Juhee Han, Dong-Hoe Koo, Do Yeun Kim, Hee-Jun Kim, Sung Young Oh, Hee Yeon Lee, Jong Hwan Lee, Hye Sook Han, Hyerim Ha, Jin Hyoung Kang

**Affiliations:** 1 College of Pharmacy, Ewha Womans University, Seoul 03760, Korea; greeni77@gmail.com (G.B.); sjbae@ewha.ac.kr (S.B.); hanju1996@naver.com (J.H.); 2 Department of Statistics, Ewha Womans University, Seoul 03760, Korea; donghwan.lee@ewha.ac.kr; 3 Division of Hematology/Oncology, Department of Internal Medicine, Kangbuk Samsung Hospital, Sungkyunkwan University School of Medicine, Seoul 03063, Korea; dhkoo.smc@gmail.com; 4 Division of Hematology/Oncology, Department of Internal Medicine, Dongguk University Ilsan Hospital, Seoul 10326, Korea; smdkdy@hanmail.net; 5 Division of Hematology/Medical Oncology, Department of Internal Medicine, Chung-Ang University Hospital, Seoul 06974, Korea; heejun@cau.ac.kr; 6 Department of Internal Medicine, Dong-A University Hospital, Dong-A University College of Medicine, Busan 49236, Korea; drosydrosy@naver.com; 7 Division of Oncology, Department of Internal Medicine, Yeouido St. Mary’s Hospital, The Catholic University of Korea, Seoul 07345, Korea; urloved@catholic.ac.kr; 8 Department of Pharmaceutical Benefits, Health Insurance Review & Assessment, Wonju 26465, Korea; jongdal@hira.or.kr; 9 Department of Internal Medicine, College of Medicine, Chungbuk National University, Cheongju 28644, Korea; sook3529@daum.net; 10 Division of Hematology & Oncology, Department of Internal Medicine, Inha University Hospital, Incheon 400 711, Korea; ha.hyerim@snu.ac.kr; 11 Medical Oncology, Seoul St. Mary’s Hospital, The Catholic University of Korea, Seoul 07345, Korea

**Keywords:** oncology, value frameworks, country adaptation

## Abstract

This study sought to adapt the existing value framework (VF) to produce a reliable and valid Korean oncology VF. Two VFs developed by The American Society of Clinical Oncology (ASCO) and the European Society for Medical Oncology (ESMO) were selected for examination in the present study. Forward and backward translations were conducted for six high-priced drugs indicated for non-small-cell lung cancer and multiple myeloma. Inter-rater reliability was measured based on the intraclass correlation coefficient (ICC) and variation was described using the coefficient of variation. The relative weights of factors critically considered by Korean oncologists were derived following the analytic hierarchy process (AHP), and focus group interviews (FGIs) were used to obtain qualitative data regarding the applications of these two VFs in the Korean setting. The ICCs of the Korean VFs were 0.895 (0.654–0.983) for ASCO and 0.726 (0–0.982) for ESMO translations, suggesting excellent reliability for ASCO and good reliability for ESMO. AHP demonstrated that clinical benefit has the highest priority, which is consistent with the ASCO VF. The FGIs suggested that the result for AHP is acceptable and that both ESMO and ASCO VFs should be used complementarily. Although further evaluation with a larger sample size is needed, the Korean versions of ESMO/ASCO VFs are valid and reliable tools and are acceptable to Korean stakeholders, yet they should be applied with caution.

## 1. Introduction

Healthcare expenditures related to cancer comprise 1.9% to 7% of total healthcare costs in Organization for Economic Co-operation and Development (OECD) countries, with the mean monthly spending on new oncology medicines increasing up to tenfold during the past decade [1]. Despite their high prices, the values of oncology medicines in terms of clinical evidence, regarding benefits such as extending overall survival and improving quality of life, remain unclear [2,3,4]. These drugs are frequently authorized based on surrogate outcomes with less than rigorous clinical trial designs [5], resulting in poor or uncertain cost-effectiveness [6]. Although the Korean health insurance system has officially adopted economic evaluation as part of the reimbursement process [7], cancer drugs with uncertain economic values are frequently discussed due to concerns such as lack of alternatives or severity of disease [8], which raise concerns regarding financial sustainability.

Institutions such as the American Society of Clinical Oncology (ASCO), the European Society for Medical Oncology (ESMO), the Institute for Clinical and Economic Review (ICER), and the National Comprehensive Cancer Network (NCCN) have introduced value frameworks (VFs) to quantify the clinical value of oncology drugs. The ESMO—Magnitude of Clinical Benefit Scale (ESMO-MCBS) and the US ASCO—Net Health Benefit (ASCO-NHB) include separate scales for curative and noncurative disease and consider the importance of quality-of-life gains and toxicity related to treatment [9]. The ASCO-NHB was intended to facilitate shared decision-making between oncologists and patients, taking into account the financial status, treatment goals, and preferences of individual patients, and ASCO-NHB scores are continuous [9]. The ESMO framework is primarily advocated for use by policymakers and payers [9] and presents a relative ranking of clinically significant benefits [10]. Limitations of both tools include that very few clinical trials have measured quality of life, making it difficult to evaluate the corresponding subdomain shared by the two VFs [11]. Although they have different objectives, both frameworks are reliable, validated tools to evaluate the clinical benefits of new cancer treatment.

The ASCO-NHB and ESMO-MCBS have been widely used in corresponding jurisdictions, but it is not known whether these tools can be used in the context of other countries [12,13]. No previous studies have evaluated the validity of adaptations or translations of these two tools into their own language. The purpose of this study was to develop Korean versions of ESMO or ASCO VFs with the goal of applying them in the context of South Korea’s national health insurance system, in which the financial burden of expensive cancer drugs has soared recently.

## 2. Materials and Methods

### 2.1. Overview

ESMO-MCBS is a framework to evaluate the magnitude of the clinical benefit in the new treatment of solid tumors. This tool has two parts and five forms: A curative setting (Form 1) and a palliative setting (Forms 2a, 2b, and 3) with two different scales A, B, or C and 1 to 5 [14]. The ESMO-MCBS assigns categorical benefit scores to positive randomized clinical trials. Primary or secondary endpoints included in the scoring system are overall survival (OS), progression-free survival (PFS), quality of life (QoL), and treatment toxicity [9].

ASCO-VF assigns a Net Health Benefit score with four main components: clinical benefit, toxicity, bonus points (tail of the curve palliation of symptoms, quality of life (QOL), treatment-free interval (TFI)), and drug acquisition (DAC) cost per month [9]. It is intended to be applied to randomized trials but not specifically applied to trials that show statistical significance in dominance trials [14].

**Figure 1:**
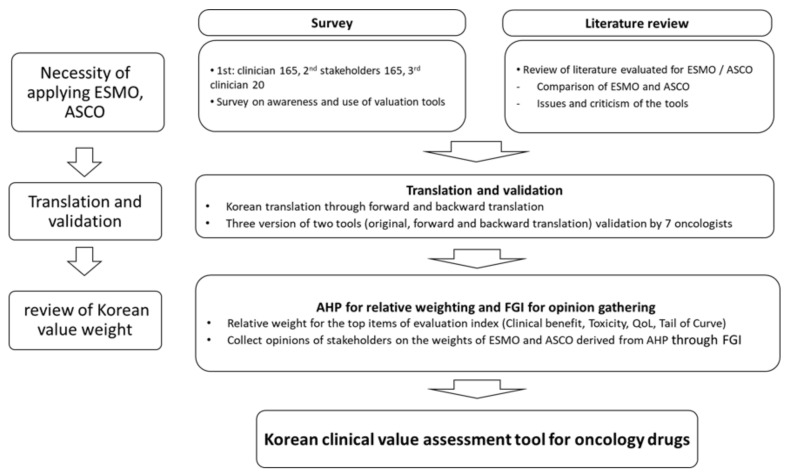
Flowchart of oncology drug value framework adoption.

As shown in Figure 1, a survey was conducted and the Korean version of the Value Framework was derived based on the survey results. Kim et al. (2020) assessed the need for adopting VFs in the Korean setting and found more than 75.9% of 166 respondents believed that both the ESMO-MCBS v1.1, and the Value Framework ASCO-NHB v2 should be considered for adaptation, to compensate for differences between the two frameworks [15]. Most experts have suggested that rather than develop a new tool from scratch within a limited timeline, adapting validated, reliable tools, such as ESMO-MCBS and ASCO-NHB, to the Korean context is more feasible. Therefore, in this study, we examined how to adapt and apply these two tools in the Korean context.

### 2.2. Translation

We conducted forward (English to Korean) and backward (Korean to English) translations of the two frameworks, which have been previously used to culturally adapt valuation tools for measure health [16]. First, we translated ESMO-MCBS v1.1 and the ASCO-NHB v2 into Korean. Two independent researchers (G.B., S.B.) produced the translations and two oncologists (D.Y. K., H.H.) and one biostatistician (D.L.) reviewed the validity of the translations. Instead of using sentences that explained the arithmetic equations in words, the formulae were introduced in the Korean translated version to reduce confusion and increase consistency across evaluators. The terms that need to be clarified were described uniformly by referring to the “Cancer Clinical Trial Guidelines’’ of the Korea Food and Drug Safety Evaluation Institute [17]. Finally, a bilingual oncologist performed a backward translation of the Korean translation into English to examine the validity of the translation.

### 2.3. Panelists

Twenty panelists including twelve physicians, and eight non-physician researchers were selected for testing validity and reliability. Following Zou (2012), we calculated the required sample size and number of panelists for achieving the statistical power at 80% [18]. For investigating the six drugs, eight panelists were enough. The Korean Chemotherapy Research Society recommended twelve physicians and the International Society for Pharmacoeconomics and Outcomes (ISPOR) Korea chapter recommended twelve non-physician experts. Finally, a total of 12 physicians and eight non-physician professionals made up the panel to evaluate the translated tools. Specifically, seven panelists (five oncologists and two non-physician researchers) were included for the original version, five panelists (three oncologists and two non-physician researchers) for the translated version, and eight panelists (four oncologists and four non-physician researchers) responded. Non-physician researchers were doctorate-level health services researchers who had experience in oncology research.

### 2.4. Drugs

For validation, six anticancer drugs were selected and evaluated using the original version, Korean, and backward translation versions of the VFs. These six drugs were selected based on indications, malignancies, mechanisms, patient needs, and high prices. They included one targeted therapy for non-small-cell lung cancer, three cancer immunotherapies, and two treatments for multiple myeloma. A targeted therapy for non-small-cell lung cancer was selected because it showed the highest sales value in 2020. Three cancer immunotherapies were chosen since their off-label use can be reimbursed by the National Health Insurance (NHI) after being reviewed by the multidisciplinary committee of hospitals and Health Insurance Review & Assessment (HIRA), and used based on the risk-sharing agreements. Lastly, two treatments for multiple myeloma that pose a significant financial burden on NHI were selected since their sales value showed a substantial increase in 2020 compared to 2019. The clinical values of those drugs are of great interest to the Korean society and the NHI based on the disease severity and budget impact they entail.

### 2.5. Reliability and Validity of ASCO v2.0 and ESMO-MCBS v1.1

As in Bentley’s validity study [19], the panelists evaluated each selected drug once using each value framework. We provided an evaluation guide and a full-text clinical research paper to ensure that all evaluators calculated scores and levels based on the same clinical research papers. In this study, different panelists evaluated the original version, Korean translation version, and backward translation version to confirm the reliability and validity of the Korean translation. Their discussions contributed to the use of formulas or separate guides for unclear or misleading parts of the Korean versions of value frameworks based on their evaluation experience. The “net-health benefit” scores of the ASCO range from −20 (worst) to 180 (best), and the ESMO scores range from 1 (worst) to 5 (best). ASCO value frameworks were determined based on clinical efficacy, toxicity, effects on long-term survival, palliation, quality of life, and treatment-free interval.

### 2.6. Analysis

For each drug, the mean and standard deviation (SD) of total and subdomain scores in the ASCO tool were calculated. For the ESMO-MCBS tool, only the total level score was assessed. The distributions of the evaluators’ scores were described using coefficients of variation (CV), and the consistency of the results was evaluated using Bland–Altman plots and the intraclass correlation coefficient (ICC). A Bland–Altman plot is a scatter plot that calculates the mean and the difference for each side of the two sets of measurements for the same subject, then uses the mean as the *x*-axis and the difference as the *y*-axis. This method is recommended to assess discrepancies between measurements made by two different methods, as well as for evaluating repeatability and reproducibility [20]. ICC (2, k) values were analyzed to determine inter-rater reliability [21].

ICC ranges from 0 to 1, with values less than 0.40 indicating low reliability, while 0.40–0.59 represents fair reliability, and 0.60–0.74 represents good reliability. When the value is greater than 0.75, it can be interpreted as indicating excellent reliability [16]. When analyzing the ICC, the respondents used a two-way random-effects model that assumed that they were random samples and a 95% confidence interval. The ASCO VF was composed of three subdomains (clinical benefit, toxicity, and bonus point), and thus three ICC values for each subdomain were measured. In addition, we verified the reliability of the final scorers using ASCO and ESMO tools in the Korean translation and backward translation. In the Table 1, values marked with † are assumed to be 0, which means that the actual ICC value is very low [20].

### 2.7. Analytic Hierarchy Process (AHP)

The analytic hierarchy process (AHP) was conducted to derive priorities and relative weights of clinical benefit, toxicity, and bonus categories including quality of life and additional clinical improvements, and costs, which are categories considered in the ESMO and ASCO frameworks [22]. Fifteen oncologists from the Korean Chemotherapy Research Society participated in this analysis. The consistency index (CI) was calculated to determine whether there were any inconsistent responses, but there were no cases of inconsistency among the respondents [23]. In cases of omitted responses, the value was later requested via e-mail and telephone.

### 2.8. Focus Group Interview (FGI)

The FGI took place once in December 2019. The participants in the FGI consisted of four hematological oncologists, two pharmaceutical association executives, and two representatives of patient organizations. It entailed 90 min group discussions that were held on the following topics: (1) AHP results for weight and priority related to clinical benefit, toxicity, and bonus considerations including quality of life and cost, (2) perceptions and attitudes toward the oncology drug value assessment tool, and (3) overall opinions and comments about applying the Korean versions of ESMO/ASCO in the context of the Korean health system.

## 3. Results

### 3.1. Validity of Translation

Table 1 shows the validation scores of the original, forward, and backward translation versions according to the mean, standard deviation, and coefficient of variation. The ICC of the total score was 0.899 (95% CI 0.695–0.984) in the original version, 0.895 (95% CI 0.654–0.983) in the forward translation version, and 0.930 (95% CI 0.792–0.989) in the backward translation version for ASCO-NHB, and 0.749 (95% CI 0.007–0.982) in the original version, 0.726 (95% CI 0†–0.982) in the forward translation version, and 0.900 (95% CI 0.604–0.993) in the backward translation version for ESMO-MCBS (Table 1). All six versions had excellent reliability. However, the ICC of the clinical benefit score in the original version (0.620) was lower than that of the translated (Korean) version (0.973), while the ICC of the translated (Korean) version (0.407) was lower than that of the original version (0.928). The ICC of the bonus point in original and forward translation versions showed good reliability (0.791 and 0.646, respectively), yet it was low compared to that of the backward translation version (0.557).

The coefficient of variation showed that the toxicity score had the widest variance in all six versions (from −4.55 to −13.81), followed by the bonus point (drug D from 0.73 to 1.12). Less than 30% of the variation was found in forward and backward translated versions of the clinical benefit. The variances for other drugs were similar across versions.

### 3.2. Relative Weights of Evaluation Framework Variables

Fifteen members of the Korean Chemotherapy Research Society responded to questions about the relative importance of factors considered when prescribing chemotherapy drugs and immune-cancer drugs through AHP Technic. Clinical benefit was regarded as the most important factor when prescribing chemotherapy drugs and immune-cancer drugs (Table 3). However, the perceived clinical benefit of the immune-cancer drug (0.54) was about 2.6 times more important than toxicity (0.21). The clinical benefit (0.51) of general oncology drugs was 1.9 times more important than toxicity (0.27). The importance of cost-effectiveness was 3.6 times higher for immune-cancer drugs, and 4.25 times higher for chemotherapy drugs. The cost of immune-cancer drugs was considered more important than those of general oncology drugs. The ease of treatment and nonclinical factors were similar for both immune-cancer drugs and chemotherapy drugs. We explained the ASCO and ESMO tools in detail to our panel, and then examined the relative weights of the ASCO indicators based on the premise that both tools were fully understood. As a result, the clinical benefit was found to be about 2.7 times more important than toxicity as shown in Table 3. Treatment-free intervals and cost were also considered similarly important, with clinical benefits considered five times and toxicity considered 1.7 to 1.9 times more important than treatment-free intervals and cost [Table 2].

### 3.3. Opinions of Stakeholders

Stakeholders deeply agreed with the need for using VFs that could quantify the value of oncology drugs for decision-making. In particular, clinicians (oncologists) and patient groups responded that validated, objective tools for evaluating value should be used because of high clinical uncertainties regarding the benefits of cancer drugs while their prices are soaring. Clinicians and patients suggested that the results for AHP are not surprising and agreed regarding the relative importance of clinical benefit in the Korean setting.

Clinical experts were concerned about scientific arguments about imperfections of VFs and stated that both ASCO and ESMO frameworks should be implemented complementarily due to their pros and cons. Clinicians also suggested that adopting already-developed frameworks such as ESMO or ASCO is a feasible option, given that the factors and relative weights considered in the ASCO or ESMO frameworks have been rigorously examined, validated, and updated. They added that these tools may not be applicable to multiple myeloma drugs. Clinicians emphasized that considerable training and discussion are required to apply these tools and pointed out that proper evaluation cannot be expected unless clinicians are fully educated regarding their contents. Although the clinicians fully embraced the use of ASCO-NHB and ESMO-MCBS, they suggested a very careful, phased approach is needed if these tools were applied in the contexts of pricing and reimbursement. The patient groups placed more emphasis on clinical benefits than on costs, thanks to the extensive health insurance coverage in Korea [Table 3].

## 4. Discussion

In this adaptation study, we validated the Korean versions of VFs using forward/backward translation, examined the reliability of the Korean versions using ICC, investigated Korean weights for each variable using AHP, and collected stakeholders’ opinions through FGIs and clinician panels. We demonstrated that the translated versions of ESMO-MCBS and ASCO-NHS are reliable and acceptable in the Korean setting and suggest that countries other than the U.S. or those in the European Union should consider using already-developed tools. While there are differences between the ASCO-NHB and ESMO-MCBS, both frameworks seek to provide clear and proven approaches to assessing clinical benefits and analyzing clinical data. ESMO and ASCO have been revised based on rigorous reviewing and collection of opinions with input from many stakeholders [9,10,24,25].

Among the three versions of ASCO NHB scores, the backward translation showed slightly higher consistency, which could be attributable to the fact that the respondents used to test the backward translation mostly participated in the AHP and FGI and had previously been educated about the two frameworks. However, all three versions showed good reliability with ICC values of 0.6 to 0.74, while a value over 0.75 indicates excellent reliability. It also can be found in Appendix A. In addition, the Korean versions introduced arithmetic equations to evaluate clinical benefit, such as hazard ratio (HR) and overall survival (OS), whereas the original versions explained these concepts in words. This helps explain the relatively high ICC for the translated (Korean) versions (0.973 vs. 0.620). However, relatively poor ICC scores for toxicity and bonus points in the Korean versions (0.407) remain as limitations of our translated tools. Compared to other values, the toxicity score is usually smaller in absolute number, so the variance may seem larger. However, the low reliability of toxicity scores is consistent with the results of previous studies [20,26] and may be explained by the characteristics of the ASCO tool that applies both low and high grades, unlike ESMO, which only applies high grades of 3–4 [9,24]. Good clinical studies that clearly report grade 1, 2, 3, and 4 toxicity are limited [27], and subjective judgments may be involved in the process of evaluation. Forward-version respondents may also have a poor understandings of toxicity score calculation. However, even if their understanding was poor, it is more meaningful that the clinical benefit scores were consistent.

In previous comparative studies of these two tools, the results showed low to modest correlations between earlier versions of the two tools [27]. However, in updated studies, the correlations between the two tools increased [11,13,20,27,28,29]. The ICC of final scores of the ASCO and ESMO tools of all evaluators of the Korean translation were 0.895 for ASCO-NHB and 0.726 for ESMO-MCBS, consistent with the previous studies [20].

Our results should be interpreted with caution due to the small sample sizes used for the reliability test, AHP, and FGIs. The ASCO and ESMO Korean versions developed in this study should be developed continuously in order to provide more precise guidelines to foster objectivity and consistent evaluation. In addition, several validity tests should be used to improve the reliability of the evaluation. Further analyses should be performed due to the insufficient samples used to validate the tools in the current study. Future researchers should keep in mind that experts in the field with low knowledge and understanding of the tools may find it difficult to evaluate them and should make efforts to increase the awareness of value assessment tools. Moreover, the application of the VFs in the Korean context should be discussed further in future research.

Weights derived from AHP in the Korean context were particularly important in terms of evaluating clinical benefit, and clinicians considered clinical benefit (0.4) to be the most important factor when determining clinical value. However, oncologists were also concerned about toxicity (0.15) and quality of life (0.14) when they considered the ASCO framework. Further research should determine whether the scores of variables calculated as bonus items in the ASCO VF are appropriate in the Korean context and for evaluating the clinical values of oncology drugs used by Korean clinicians.

The application of these tools in the context of insurance coverage should be approached very carefully. As can be seen from the FGI results, the focus group fully agreed to quantify clinical value academically for clinical practice. However, there are also concerns from various stakeholders in the contexts of insurance coverage. We think it can be used as one of the various criteria for post-reimbursement evaluation.

## 5. Conclusions

This paper describes the development of the first localized versions of ASCO/ESMO VFs and validates a Korean version of the ESMO/ASCO VFs. We demonstrate that they are valid, reliable tools and are acceptable to Korean stakeholders based on the six high- priced oncology drugs. Because this study is preliminary, the Korean VFs presented in this study should be developed continuously in order to provide more precise guidelines.

## Figures and Tables

**Table 1:** Validation scores for six drugs.

Drug	Original Version				Forward Translation				Backward Translation			
	ASCO		ESMO		ASCO		ESMO		ASCO		ESMO	
	Mean ± SD	CV	Mean ± SD	CV	Mean ± SD	CV	Mean ± SD	CV	Mean ± SD	CV	Mean ± SD	CV
Overall												
ICC	0.899 (0.695–0.984)		0.749 (0.007–0.982)		0.895 (0.654–0.983)		0.726 (0 ^†^–0.982)		0.930 (0.792–0.989)		0.900 (0.604–0.993)	
A	78.63 ± 24.44	0.31	3.71 ± 0.49	0.13	90.27 ± 8.48	0.09	4.00 ± 0.00	0.00	87.07 ± 11.73	0.13	3.88 ± 0.83	0.21
B	75.72 ± 14.22	0.19	3.57 ± 1.40	0.39	73.29 ± 12.48	0.17	3.80 ± 1.64	0.43	78.66 ± 17.33	0.22	4.75 ± 0.71	0.15
C	59.84 ± 17.07	0.29	3.29 ± 1.60	0.49	58.36 ± 14.30	0.25	3.80 ± 1.64	0.43	57.78 ± 9.46	0.16	3.38 ± 1.41	0.42
D	49.55 ± 26.35	0.53	1.86 ± 0.38	0.20	42.23 ± 17.76	0.42	1.80 ± 0.45	0.25	50.60 ± 19.62	0.39	2.25 ± 0.46	0.20
E	39.71 ± 11.04	0.28		-	39.95 ± 7.88	0.20		-	39.74 ± 11.08	0.28		-
F	54.38 ± 17.36	0.32		-	60.16 ± 14.04	0.23		-	70.24 ± 15.20	0.22		-
Clinical benefit												
ICC	0.620 (0 ^†^–0.939)				0.973 (0.910–0.996)				0.973 (0.921–0.996)			
A	51.29 ± 22.15	0.43		-	60.00 ± 6.16	0.10		-	62.70 ± 9.54	0.15		-
B	41.57 ± 9.29	0.22		-	38.20 ± 6.26	0.16		-	38.24 ± 5.55	0.15		-
C	35.00 ± 15.87	0.45		-	29.00 ± 0.00	0.00		-	31.78 ± 7.01	0.22		-
D	33.57 ± 17.39	0.52		-	27.00 ± 0.00	0.00		-	30.35 ± 4.62	0.15		-
E	29.04 ± 12.07	0.42		-	24.66 ± 4.37	0.18		-	26.20 ± 4.29	0.16		-
F	34.46 ± 7.99	0.23		-	32.24 ± 8.54	0.26		-	44.83 ± 4.56	0.10		-
Toxicity												
ICC	0.928 (0.781–0.988)				0.407 (0 ^†^–0.906)				0.823 (0.477–0.971)			
A	4.16 ± 5.27	1.27			1.69 ± 1.01	0.60		-	1.62 ± 1.12	0.69		-
B	9.89 ± 9.08	0.92			7.13 ± 9.43	1.32		-	11.67 ± 6.84	0.59		-
C	3.41 ± 6.77	1.99			0.36 ± 4.97	13.81		-	4.75 ± 7.96	1.68		-
D	3.12 ± 3.62	1.16			1.23 ± 1.62	1.32		-	4.37 ± 6.38	1.46		-
E	1.12 ± 4.39	3.92			−0.30 ± 1.68	−5.60		-	−2.71 ± 7.21	−2.66		-
F	−1.60 ± 7.28	−4.55			−0.20 ± 2.65	−13.25		-	−0.81 ± 4.39	−5.42		-
Bonus point												
ICC	0.781 (0.338–0.965)				0.646 (0 ^†^–0.944)				0.557 (0 ^†^–0.928)			
A	23.14 ± 11.71	0.51			28.40 ± 9.21	0.32		-	22.75 ± 5.75	0.25		-
B	28.57 ± 12.15	0.43			34.00 ± 8.94	0.26		-	28.75 ± 13.56	0.47		-
C	20.00 ± 16.07	0.80			27.00 ± 13.04	0.48		-	21.25 ± 13.56	0.64		-
D	12.86 ± 14.96	1.16			14.00 ± 16.73	1.20		-	16.25 ± 11.88	0.73		-
E	12.86 ± 7.56	0.59			16.00 ± 5.48	0.34		-	16.25 ± 5.18	0.32		-
F	22.00± 13.27	0.60			28.80 ± 7.01	0.24		-	26.25 ± 11.63	0.44		-

ASCO: American Society of Clinical Oncology; ESMO: European Society for Medical Oncology; Mean ± SD: mean ± standard deviation; CV: coefficient of variation; ICC: intraclass correlation coefficient; ^†^: this indication was negative as a result of ICC analysis, and it was assumed that the actual ICC value was very low.

**Table 2:** Relative weights by analytic hierarchy process (AHP).

Relative Weights		Prescribing Oncology Drug	Prescribing Immuno-Cancer Drug	ASCO Variables
Clinical benefit		0.51	0.54	0.40
Toxicity		0.27	0.21	0.15
Bonus consideration	QoL	0.05	0.04	0.14
	Additional clinical improvements *	0.05	0.06	0.25
Cost		0.12	0.15	0.08

ASCO: American Society of Clinical Oncology; QoL: quality of life; * such as symptom palliation, treatment-free interval, and long-term survival.

**Table 3:** Core opinions in focus group interviews (FGIs).

Category	Core Opinions
Opinions on the relative weights used in the oncology value assessment tool	✓ Emphasis on clinical benefit is higher in Korea than in the U.S. or other countries (clinicians and patient group)
Necessity of oncology drug valuation tool	✓ Required due to uncertainties in clinical evidence (clinicians and patient group) ✓ Adopting already-developed frameworks is more feasible (clinicians)
Implementation of frameworks	✓ Both ESMO and ASCO frameworks should be considered complementarily (clinicians) ✓ Sufficient training in ASCO and ESMO are needed (clinicians)
Application of frameworks in the reimbursement system	✓ Very careful approach to interpreting results (all stakeholders)

ESMO: European Society for Medical Oncology; ASCO: American Society of Clinical Oncology.

**Figure A1:**
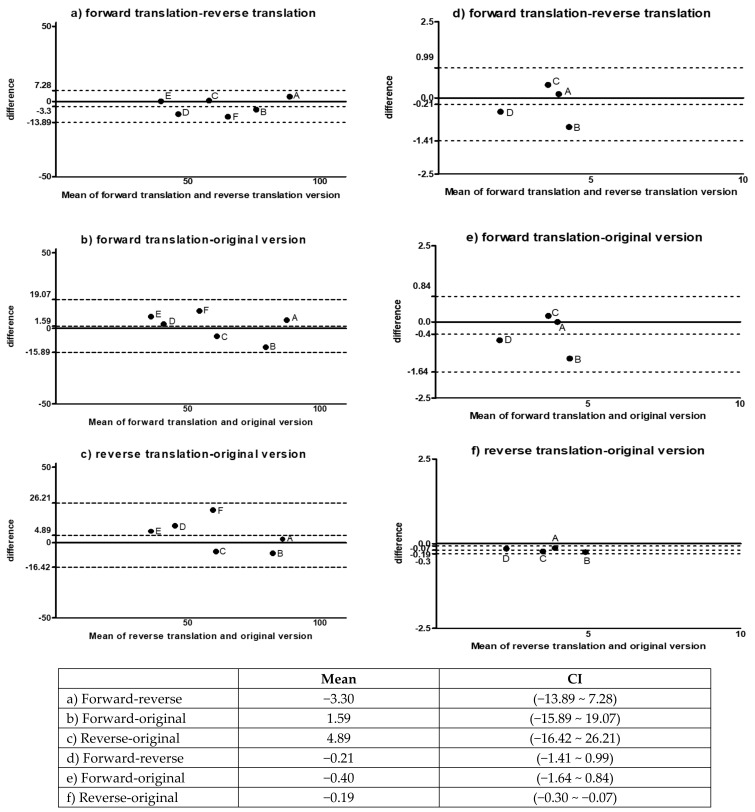
Bland–Altman plots for ASCO and ESMO evaluation. Bland–Altman plot for (**a**) forward translation and reverse translation, (**b**) forward translation and original version, (**c**) reverse translation and original version used for ASCO evaluation. Bland–Altman plot for (**d**) forward translation and reverse translation, (**e**) forward translation and original version, (**f**) reverse translation and original version used for ESMO evaluation. The median dotted lines parallel to the *x*-axis indicate the mean. The above and below dotted lines indicate the 95% confidence limits. A~F are Clinical Value assessment result of the six drugs including one targeted therapy for non-small cell lung cancer, three cancer immunotherapies, and two treatments for multiple myeloma

## Data Availability

Not publicly available
